# Effect of Orexin receptor antagonists for patients with Lewy bodies: Lemborexant do not make worse cognitive function and REM sleep Behavior Disorder in DLB

**DOI:** 10.1002/alz70857_097973

**Published:** 2025-12-24

**Authors:** Soichiro Shimizu, Shoya Inagawa, Ryo Yamamoto, Yuta Inagawa, Naoto Takenoshita

**Affiliations:** ^1^ Tokyo Medical University, Shinjuku‐ku, Tokyo, Japan; ^2^ Tokyo Medical University, Tokyo, Japan

## Abstract

**Background:**

In daily clinical setting, we often hear that clinical physician do not use orexin receptor antagonists because those induce nightmares and RBD when used in patients with DLB. We investigated the effects of Lemborexant as Orexin receptor antagonist for sleep profiles assessing by Actigraphy and cognitive function in DLB.

**Method:**

We recruited 13 probable DLB patients who take Lemborexant (5mg) for insomnia and overwent actigraphy twice before and after taking Lemborexant. The assessment of cognitive function was by Mini‐Mental state examination (MMSE). Severity of sleep disturbance were assessed by Japanese version of Epworth sleepiness scale (JESS), Japanese version of the Athens Insomnia Scale (AIS‐J) and Japanese version of REM sleep behavior disorder screening questionnaire (RBDSQ‐J). All participants wore ActiWatch (Ambulatory Monitoring Inc., NY, USA) around the wrist for 7 consecutive days. Sleep variables, including total sleep time (TST), sleep efficiency, wake state after sleep‐onset (WASO, expressed in minutes), and the number of awaking during sleep time, were calculated using Action W 2 software (Ambulatory Monitoring Inc., NY, USA) based on 7‐day records.

**Result:**

Lemborexant did not improve sleep quality in patients with DLB. Moreover, there is no significant differences in cognitive function after taking Lemborexant in DLB.

**Conclusion:**

Lemborexant does not worsen cognitive function and WASO, and does not accentuate RBD, in patients with DLB.

